# Multi-modal deep learning model for bipolar depression adolescents with verbal auditory hallucinations

**DOI:** 10.3389/fpsyt.2026.1844684

**Published:** 2026-06-19

**Authors:** Qinnaer Bolatijiang, Shaohong Zou, Cheng Zhang, Chengji Wang, Jianliang Zhang

**Affiliations:** 1 Graduate School, Xinjiang Medical University, Ürümqi, Xinjiang, China; 2 Department of Clinical Psychology, People’s Hospital of Xinjiang Uygur Autonomous Region, Urumqi, Xinjiang, China; 3 Department of Radiology, People’s Hospital of Xinjiang Uygur Autonomous Region, Urumqi, Xinjiang, China

**Keywords:** adolescent, bipolar depression, deep learning, magnetic resonance spectroscopy, verbal auditory hallucinations

## Abstract

**Objective:**

To develop a multimodal deep learning–based classification model for adolescent bipolar depression (ABD) with verbal auditory hallucinations (AVHs).

**Methods:**

A retrospective analysis was conducted on 47 untreated ABD patients within 30 days, between January 2024 and August 2025. Comprehensive clinical data were collected, including sex, age, age at onset, years of education, and the presence of suicidal or self-harming behaviors. Based on the PANSS P3 score and the presence of AVHs, patients were divided into a hallucination group (P3 score > 3, n = 24) and a non-hallucination group (P3 score ≤ 3, n = 23). All participants underwent 1H-MRS scanning of the ventromedial prefrontal cortex (vmPFC). A multimodal deep learning model was constructed using MRS-derived features in combination with clinical parameters.

**Results:**

The model achieved an optimal classification accuracy of 71.43% on the fixed test set, as obtained by the second-fold model. This best-performing model demonstrated balanced and stable classification performance for both positive and negative samples, with precision, recall, and F1-score all reaching 0.75.

**Conclusion:**

This study proposes a novel multimodal Transformer-based framework and evaluates its effectiveness in classifying ABD patients experiencing depressive episodes with AVHs. The results suggest that the advanced model architecture, incorporating mechanisms such as bidirectional cross-attention and an Ensemble of Experts classifier, can effectively integrate heterogeneous data and achieve a test accuracy of 71.43% on a small dataset, indicating preliminary technical feasibility.

## Highlights

- Bidirectional Cross-Attention Mechanism: Enables image and table features to attend to each other, achieving genuine bidirectional information fusion.
- CBAM Attention Integration: Simultaneously captures the relative importance of channel-wise and spatial dimensions within CNNs.
- SwiGLU Activation Function: Provides greater expressive power than traditional ReLU or GELU activation functions.
- Dynamic Expert Mixing: Allows the model to adaptively select the most appropriate expert network for each input sample.
- Multi-level Feature Interaction: Introduces a secondary interaction between raw features and fused representations within the classifier.

## 1 Introduction

Bipolar disorder (BD) is a severe, recurrent, and cyclical lifelong mental illness characterized by emotional instability, fluctuating cognitive activity, and abnormal mood episodes, which collectively impose substantial social and economic burdens. According to data released by the World Health Organization in 2019, BD affects approximately 0.52% of the global population, corresponding to nearly 40 million individuals worldwide. (1). More than half of adult patients with BD report disease onset before the age of 20, with some cases emerging prior to 13 years of age. (2). Bipolar depression (BD-Dep), typically defined as a major depressive episode occurring in the context of BD (3), is one of the leading causes of disability among young adults. The onset of BD-Dep during adolescence profoundly affects multiple aspects of life, including academic achievement, interpersonal relationships, and daily functioning, and is also associated with an increased risk of self-harm and suicide (4, 5).

Psychotic symptoms are also common among adolescents with BD-Dep, with reported prevalence rates ranging from 16% to 87.5% in this population (6). Among these symptoms, auditory hallucinations are the most prevalent, with approximately 37% of ABD experiencing them (7). More specifically, AVHs refer to the perception of clearly articulated, meaningful speech in the absence of external auditory stimuli. The content of these voices is predominantly negative and self-referential, often involving insults, mockery, or commanding statements (8). Pediatric and adolescent patients with BD who present with psychotic symptoms tend to experience a more protracted disease course, higher relapse rates, greater symptom severity, longer hospitalizations, poorer social functioning, and substantial difficulty achieving full remission, ultimately leading to unfavorable prognostic outcomes (9).

Magnetic Resonance Spectroscopy (MRS) is currently the only noninvasive magnetic resonance–based technique capable of detecting *in vivo* metabolic changes at the cellular level without causing tissue damage. It leverages the principles of nuclear magnetic resonance and chemical shift effects to generate spectral peaks at different frequencies (10). Deep learning (DL), a subfield of machine learning (ML), comprises algorithms based on multilayer neural networks that can automatically learn complex feature representations through successive nonlinear transformations. These models can be trained on large-scale datasets without reliance on manually engineered feature extraction procedures (11, 12).

In recent years, DL has advanced rapidly in the field of psychiatric disorders, evolving from approaches based on single data types to the development of novel diagnostic and predictive models that leverage multidimensional data and multimodal processing strategies. In 2021, Rahaman et al. proposed a multimodal deep learning framework that captures interactions among latent features by fusing data from different modalities and evaluating their complementary contributions to schizophrenia representation. This study was the first to integrate genomic information with structural and functional magnetic resonance imaging biomarkers for schizophrenia prediction (13). In addition, DL-based models have been applied to assess the emotional states of social media users, demonstrating superior performance over traditional machine learning models in complex language processing tasks (14).The present study aims to develop and evaluate an advanced multimodal deep learning model capable of integrating quantitative tabular data (e.g., metabolite ratios and clinical variables) with qualitative waveform image data derived from MRS to enable automated and accurate classification of hallucination symptoms. The primary objective is to validate the feasibility of multimodal data fusion strategies for classification tasks and to explore the potential biological relevance of model-dependent features in psychiatric research, thereby providing data-driven insights for the early identification of ABD patients with AVHs.

## 2 Methods

### 2.1 Participants

A total of 47 ABD patients were recruited from the outpatient and inpatient services of the Department of Clinical Psychology at the People’s Hospital of Xinjiang Uygur Autonomous Region between January 2024 and August 2025.

The inclusion criteria were as follows: (1) meeting the diagnostic criteria for a major depressive episode in BD according to the DSM-5; (2) a total score > 24 on the 24-item Hamilton Depression Rating Scale (HAMD) and a total score < 7 on the Young Manic Rating Scale (YMRS); (3) confirmation of diagnosis by at least two associate chief psychiatrists or senior clinicians; (4) provision of written informed consent by both participants and their legal guardians; (5) age between 12 and 18 years; and (6) right-handedness.

The exclusion criteria were as follows: (1) severe physical illnesses; (2) endocrine disorders that may contribute to psychiatric symptoms (e.g., hyperthyroidism, hypothyroidism, hypopituitarism, hyperparathyroidism, hypoparathyroidism, diabetes mellitus, adrenal dysfunction, and pheochromocytoma); (3) other psychiatric disorders (e.g., autism spectrum disorder, anorexia nervosa, bulimia nervosa, and learning disabilities); (4) primary or comorbid diagnoses defined by the DSM-5, including somatic symptom disorder, dementia, schizophrenia, delusional disorder, and substance use disorders; (5) contraindications to MRI, such as metal implants (e.g., screws, plates, or dental prostheses), pacemakers, or claustrophobia; (6) receipt of specific treatments within the previous month, including electroconvulsive therapy, psychotropic medications, or structured psychotherapy; and (7) neurological conditions, such as epilepsy, a history of severe trauma (particularly traumatic brain injury), or coma lasting longer than 5 minutes.

Among the participants, 24 presented with AVHs, 23 did not. All individuals were independently diagnosed by at least two associate chief psychiatrists or senior clinicians and were screened strictly according to the predefined inclusion and exclusion criteria. Clinical assessments were conducted using the PANSS, HCL-32, and HAMD scales. Participants were assigned to the verbal auditory hallucination (VAH) group if they met all of the following criteria: PANSS P3 ≥ 3 (with verbal auditory content), HCL-32 < 14, and a 24-item HAMD score ≥ 35. Participants were assigned to the non-hallucination group if they met all of the following criteria: PANSS P3 < 3, HCL-32 < 14, and a 24-item HAMD score ≥ 35.

Before adolescents and their parents decided whether to participate in the study, the purpose, content, and procedures were fully explained to both parties, and they were informed that all results would be used exclusively for academic research. Participation was entirely voluntary, and written informed consent was obtained from both the adolescents and their parents prior to enrollment. Participants were informed of their right to withdraw from the study at any time without penalty. Questionnaire data were collected through one-on-one interviews conducted by trained researchers in a quiet ward setting. Both verbal and written explanations were provided before data collection, and informed consent was reconfirmed. Participants completed the questionnaires independently. When questions arose, researchers offered clarification by explaining the original intent of the items in a neutral and nonjudgmental manner.

In addition, rigorous quality control procedures were implemented throughout the research process. These measures included the following: (1) all psychiatrists involved in the study received standardized training to ensure consistency in administering general information questionnaires, clinical scale assessments, and diagnostic criteria; (2) ^1^H-MRS examinations were conducted by radiologists with the rank of associate chief physician or above, all of whom underwent unified training to ensure accuracy and consistency in brain region localization and imaging data processing; (3) data obtained from systematic measurements were regarded as final, and all results were subjected to quality control review by specialized radiologists at the level of associate chief physician or higher; (4) strict adherence to predefined inclusion and exclusion criteria was maintained during participant recruitment, and quality control was enforced across all stages—including questionnaire coding, data entry, and data cleaning—to promptly identify and resolve issues, clarify procedures, and ensure standardized documentation and preservation of original records; and (5) a fixed stratified data partitioning strategy was applied during model development, in which samples were divided into a fixed test set and a training set, ensuring that class distributions in each training and validation fold were consistent with the overall dataset.

### 2.2 Collection of clinical data and assessment of patients

Demographic and clinical information was collected using custom-designed case report forms, including age, sex, age at onset, disease duration, and the presence of suicidal or self-injurious behaviors.

### 2.3 Image acquisition and data processing

^1^H-MRS was performed using an Ingenia 3.0T MRI scanner (Ingenia, Philips Healthcare, the Netherlands) equipped with a 15-channel phased-array head coil. Conventional structural MRI sequences, including T1-weighted imaging, T2-weighted imaging, and T2 fluid-attenuated inversion recovery (FLAIR), were acquired to exclude organic brain lesions. Subsequently, single-voxel ^1^H-MRS acquisition was conducted. The voxel of interest in the ventromedial prefrontal cortex (vmPFC) was set to 20 × 20 × 20 mm³, with an echo time of 35 ms, automatic shimming, and a total acquisition time of 10 minutes. The acquired spectral data were transferred to the ISP workstation and post-processed using Spectrum View software (ISP 7.0, Philips Healthcare, the Netherlands). After voxel localization, the peak areas of N-acetylaspartate (NAA), choline (Cho), myo-inositol (mI), glutamate (Glu), and creatine (Cr) in the vmPFC were quantified, and the metabolite ratios NAA/Cr, Cho/Cr, NAA/Cho, Glu/Cr, and mI/Cr were calculated.

### 2.4 Data processing and modeling

This study develops a multimodal Transformer model based on the PyTorch open-source deep learning framework (v2.0+), employing a bidirectional cross-attention mechanism to enable deep integration of image and tabular data. For data processing, feature engineering and structured data preprocessing are performed using NumPy (v1.24+) and Pandas (v2.0+), while image processing involves parsing and converting SVG-format medical images with OpenCV (v4.7+) and Pillow (v10.0+). Model training strictly follows standardized machine learning workflows, including data standardization, stratified K-fold cross-validation, and multidimensional performance evaluation implemented with Scikit-learn (v1.3+). Training processes and results are visualized using Matplotlib (v3.7+) and Seaborn (v0.12+). All experiments are conducted in a Python 3.8+ environment, with computational acceleration provided by CUDA on NVIDIA GPU–equipped devices, automatic fallback to CPU computation when GPUs are unavailable, and fixed random seeds to ensure experimental reproducibility.

#### 2.4.1 Data partitioning strategy

All models adopt a data partitioning scheme of stratified sampling combined with multi-fold cross-validation to ensure consistent class distribution across the training, validation, and test sets:

1. Data splitting ratioA balanced dataset is constructed using the create_balanced_split function. The test set is fixed at 7 samples (3–4 positive cases and 3–4 negative cases), and the remaining samples are assigned to the training/validation set (approximately 40 samples in total).
2. Cross-validation settings Five-fold Stratified K-Fold cross-validation is adopted. In each fold, the training set accounts for about 80% and the validation set for about 20%, ensuring that the class ratio of each fold is consistent with that of the overall dataset.
3. Performance test settings Although an independent test set is predefined, the total sample size of this study is only 47 cases. The results from merely 7 independent test samples are susceptible to individual differences and random factors, which leads to high contingency and insufficient statistical persuasiveness.

Therefore, to evaluate the final training performance of the model, all 47 samples are used for overall performance validation. This approach minimizes the result bias caused by small sample size and enables a more comprehensive and objective reflection of the model’s classification performance and practical application value.

In addition, the standardized data partitioning logic and data interfaces reserved in advance provide important implications for subsequent research expansion. After more high-quality multi-omics samples are collected in the future, the established partitioning scheme can be directly adopted for rigorous independent test set validation without reconstructing the data processing and model evaluation pipeline, laying a solid foundation for the subsequent iteration of this study.

Meanwhile, this study adopts a combined evaluation framework of 5-fold Stratified K-Fold cross-validation plus full-sample overall testing. Cross-validation can effectively verify the usability and training stability of the model. Supplemented by full-sample testing, this framework avoids the randomness of independent testing under small sample conditions and fully demonstrates the model’s generalization ability and universal performance. It forms a mutually corroborative and hierarchically supported evaluation system, which comprehensively and reliably validates the superior performance of the proposed model.

#### 2.4.2 Evaluation metric system

A multi-dimensional evaluation metric system is adopted to comprehensively assess model performance. The core metrics are as follows:

1. Classification Performance Metrics: Accuracy Precision, Recall, and F1-Score.
2. Model Reliability Metrics: Confusion Matrix, generalization gap (|Training Accuracy − Validation Accuracy|), and prediction confidence.
3. Training Stability Metrics: Mean and standard deviation of validation accuracy across each fold, as well as the convergence speed of training and validation loss.

#### 2.4.3 Model architecture details

This model integrates imaging data and clinical tabular data via an advanced multimodal fusion mechanism. It adopts a well-designed dual-stream encoder architecture to process image and tabular features separately. Deep feature fusion is subsequently performed through a Transformer-based cross-attention mechanism, and accurate classification is finally achieved using a mixture-of-experts classifier. The entire architecture fully takes into account the characteristics of medical data and the reliability requirements for clinical applications. The experimental model proposed in this study is an Enhanced Multimodal Transformer, whose main architecture consists of three components: a Feature Extractor(includes the Image Encoder and the Table Encoder), a Transformer Fusion Encoder, and a Mixture of Experts module.

##### 2.4.3.1 Feature extractor

Initially, image and tabular data are processed separately by the Image Encoder and Table Encoder, respectively.

(1)Image Encoder

**Figure 1:**
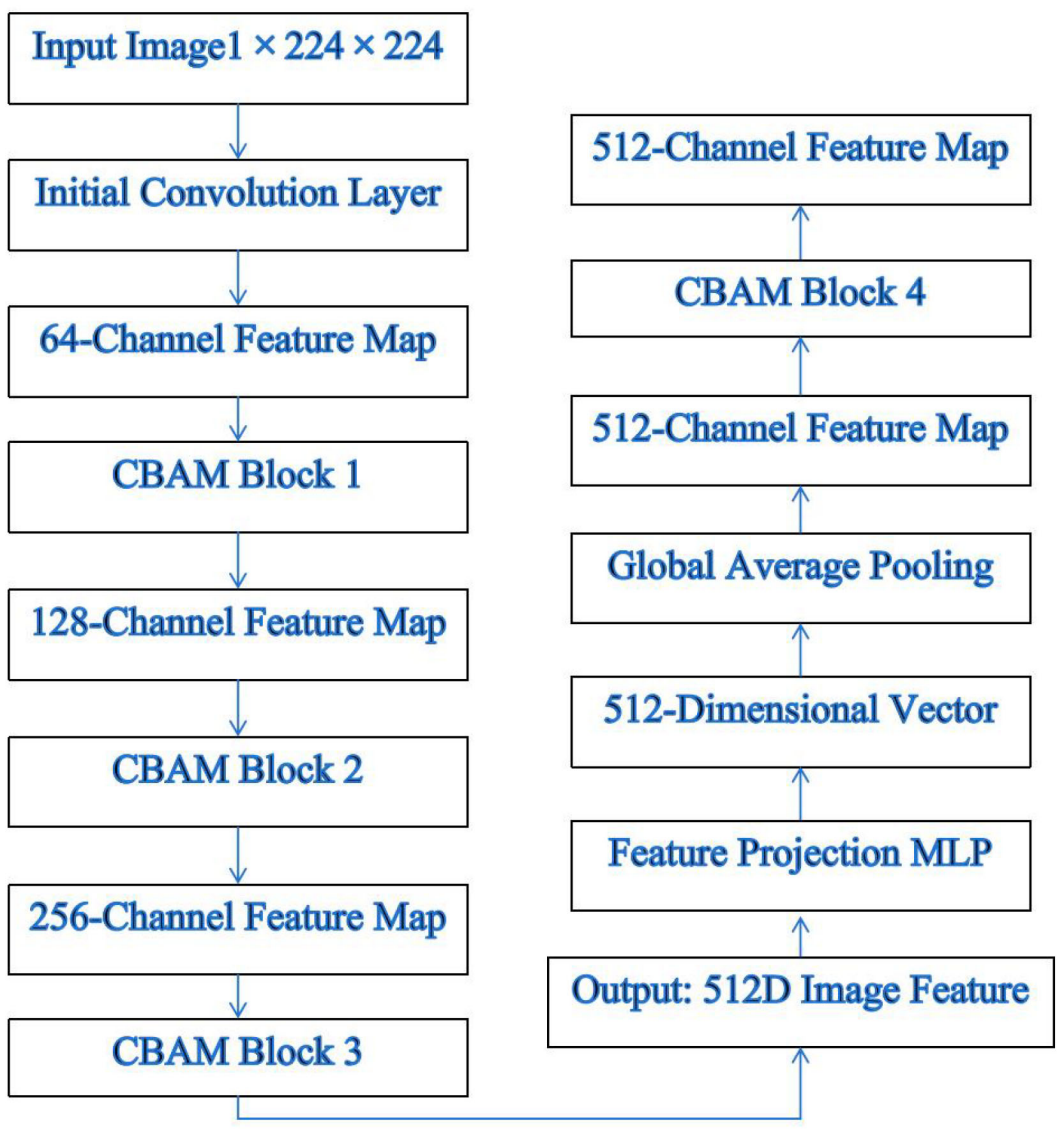
Image encoder architecture.

**Figure 2:**
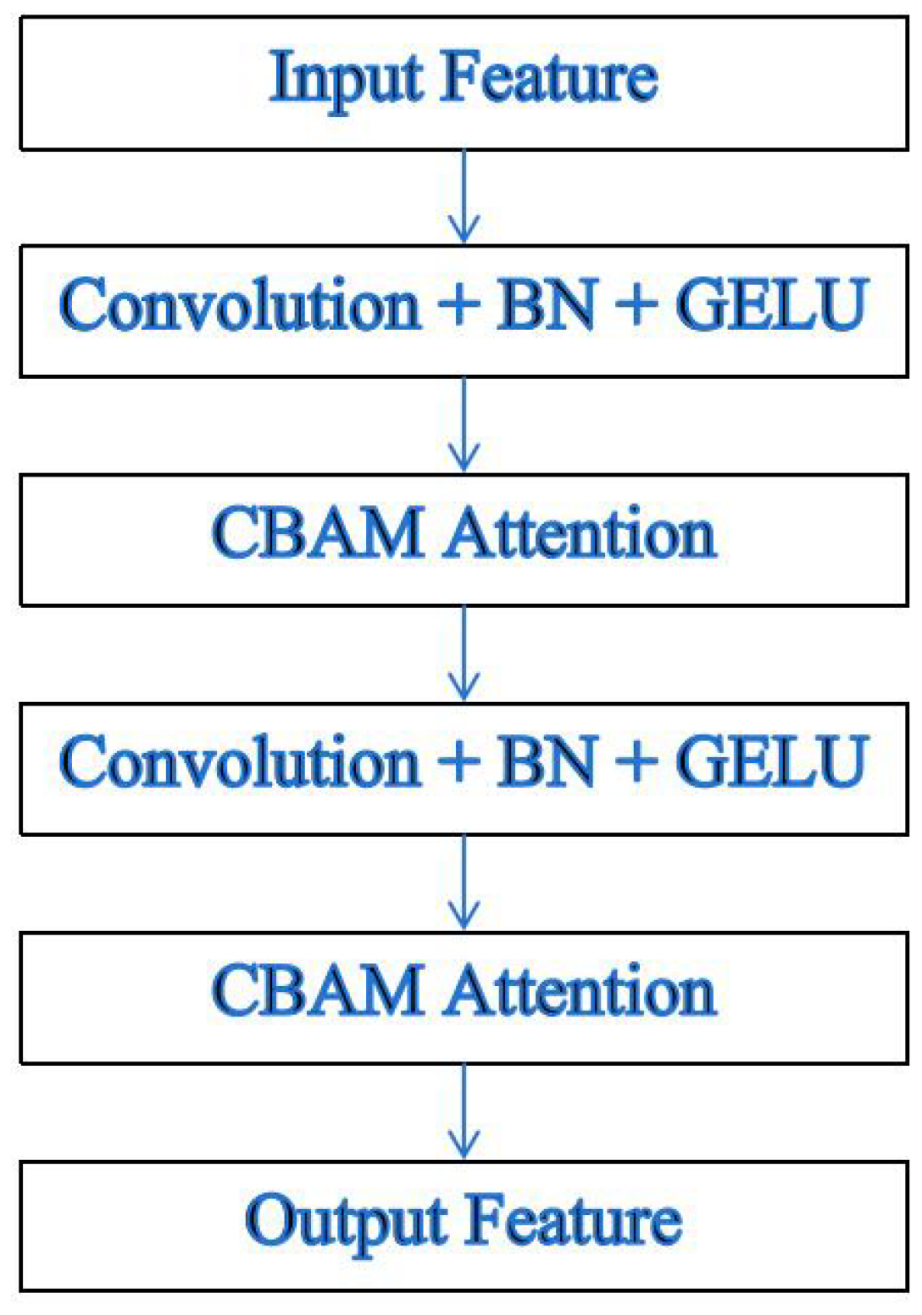
CBAM block structure diagram.

The architecture of the image encoder is shown in the **Figure 1** and **Figure 2**.

In particular, the CBAM residual block involves the CBAM attention mechanism and residual connections:

CBAM attention mechanism as shown in (Equations 1–3)

Channel attention:

(1) $$\;\;\;M_{c}(F)=\sigma (MLP(AvgPool(F))+MLP(MaxPool(F)))$$

Spatial attention:

(2) $$\;M_{s}(F)=\sigma (f^{7\times 7}([AvgPool(F);MaxPool(F)]))$$

Final Output:

(3) $$F'=M_{c}(F)⊗F\to F''=M_{s}(F')⊗F'$$

and the Residual Connection as shown in (Equation 4), Residual connection:

(4) $$y=F(x,W_{i})+x$$

(2) Table Encoder

The forward propagation of the residual block in the Table Encoder is formulated as shown in (Equation 5):

(5) $$ResBlock(x)=x+LayerNorm(GELU(W_{2}\cdot Dropout(GELU(W_{1}\cdot \;LayerNorm(x)))))$$

##### 2.4.3.2 Transformer fusion module

**Figure 3:**
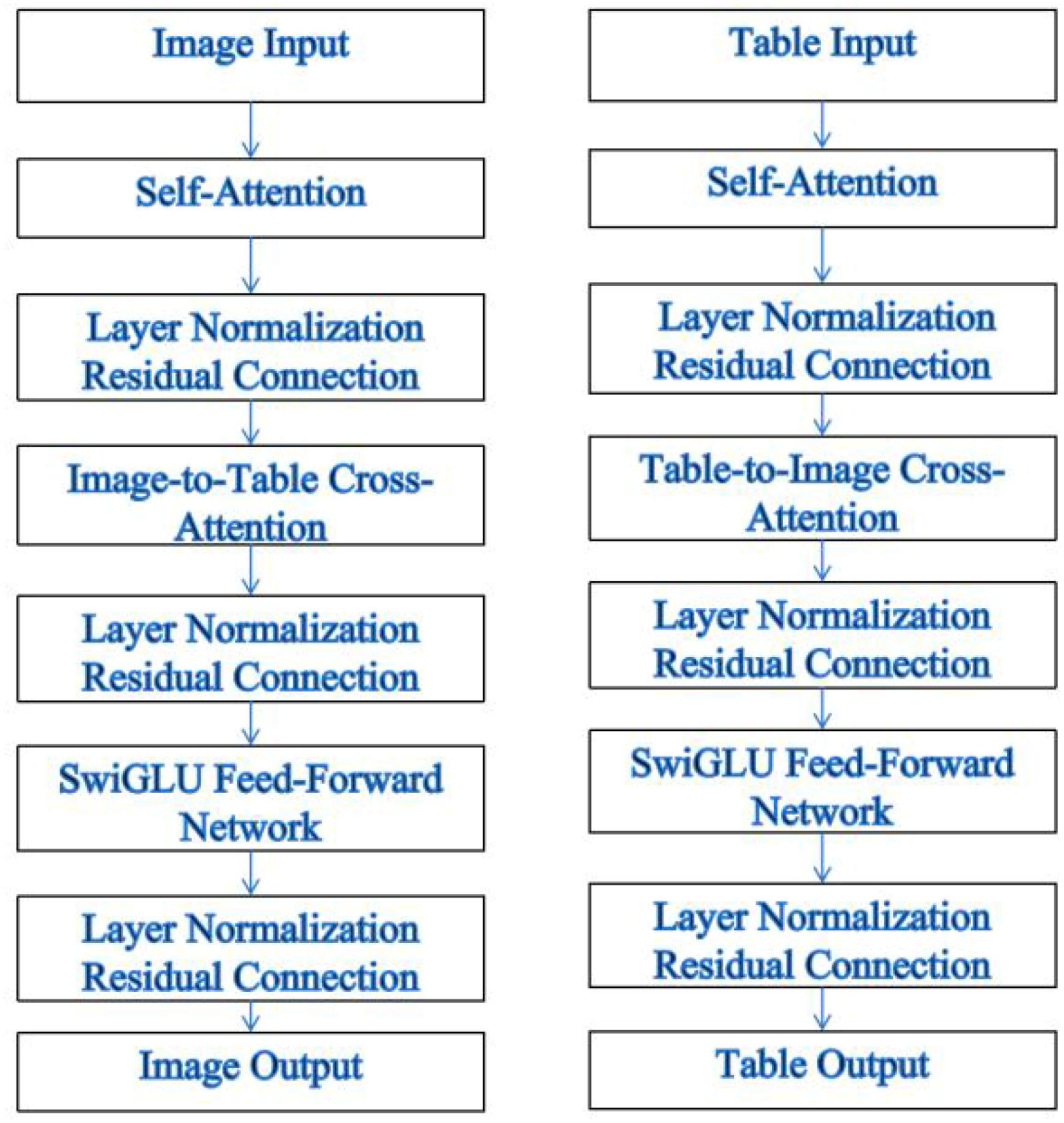
Single-layer fusion layer structure.

**Figure 4:**
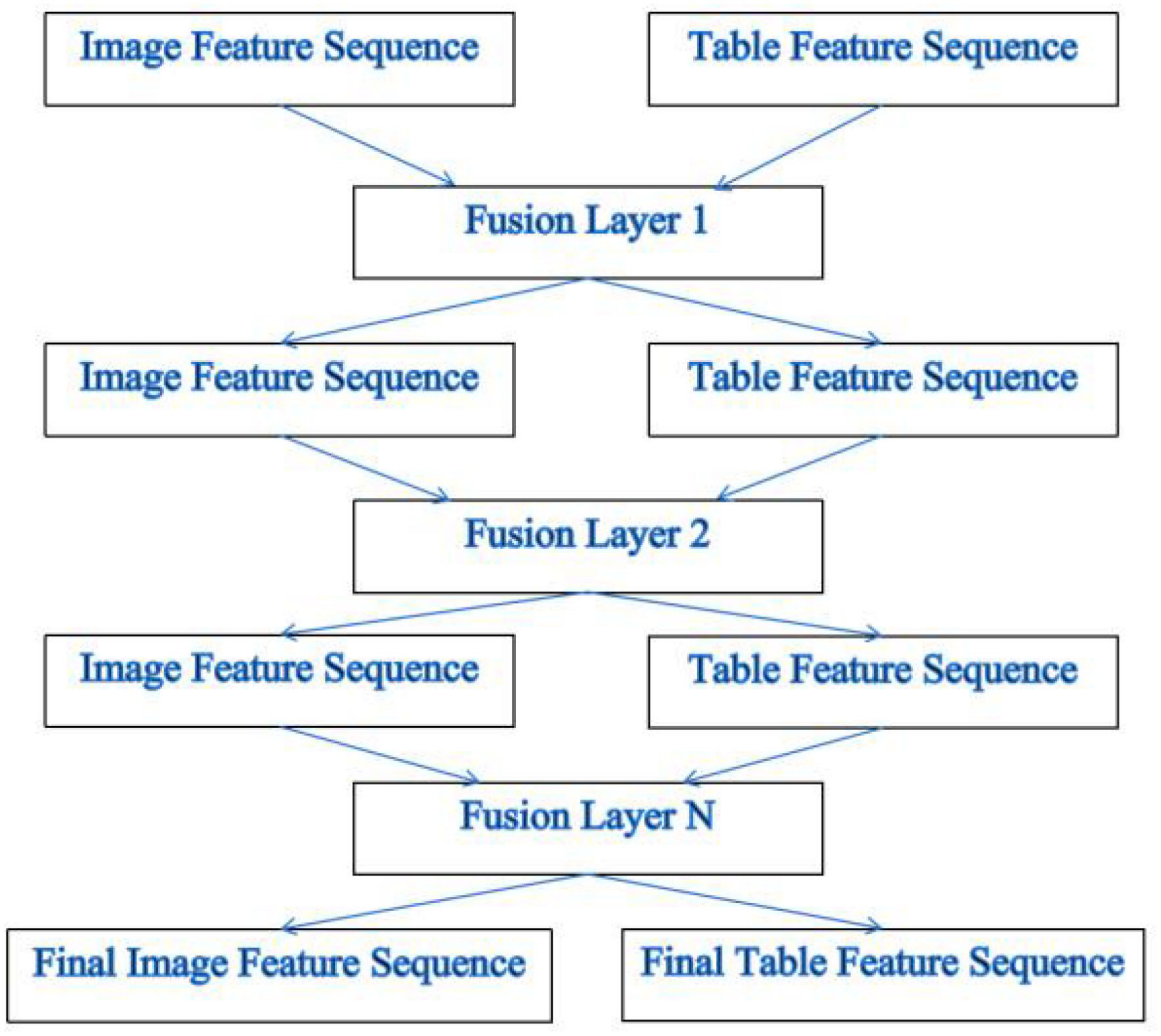
The complete fusion processing pipeline.

Furthermore, the table feature sequence and image feature sequence are fused multiple times within the fusion layers to yield the final refined table and image feature sequences (**Figures 3**, **4**).

(1)Multi Head Cross Attention as shown in (Equation 6, 7)

Attention Calculation:

(6) $$Attention(Q,K,V)=softmax(\frac{QK^{T}}{\sqrt{d_{k}}})V$$

Multi-head Projection:

(7) $$Q=W_{q}X_{q},\;K=W_{k}X_{k},\;V=W_{v}X_{v}$$

(2)Transformer Fusion Layer

SwiGLU activation function as shown in Equation 8:

(8) $$\begin{array}{c} SwiGLU(x)=x⊗\sigma (gate)\\ \;x,gate=split(Wx+b) \end{array}$$

Layer Normalization and Residual Connection as shown in Equation 9:

(9) $$\;\;\;\;\;\;\;\;\;\;\;x_{out}=LayerNorm(x_{in}+Dropout(Sublayer(x_{in})))$$

The structure of the fusion layer and the complete fusion processing pipeline is as follows:

##### 2.4.3.3 Mixture of experts classifier

**Figure 5:**
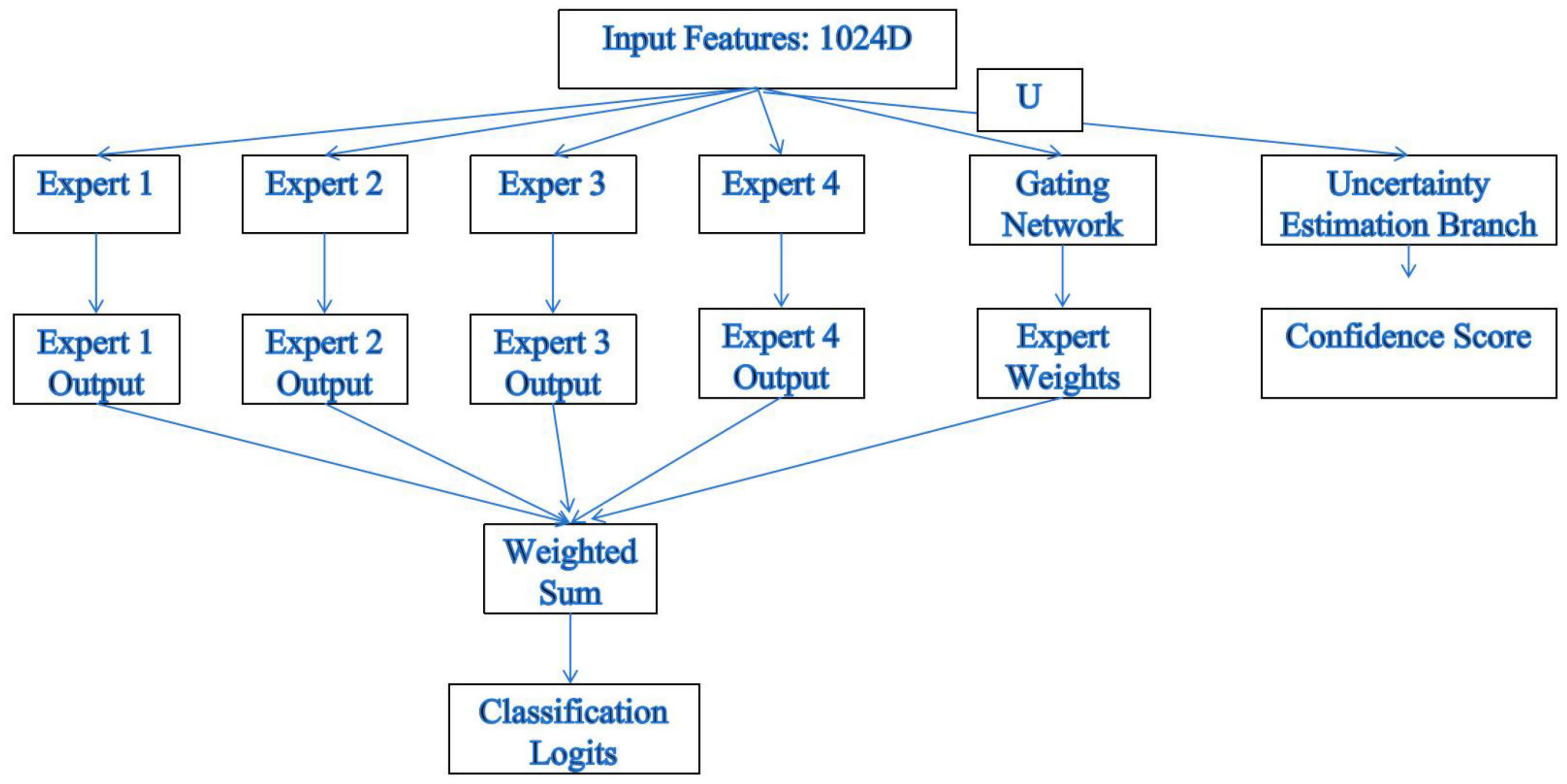
The overall architecture of the mixture-of-experts classifier.

Finally, the mixture-of-experts classifier is utilized to implement mixed output and feature interaction for all features. After weighted summation, the classification logits are derived (**Figure 5**).

Mixture-of-Experts Output as shown in Equation 10:

(10) $$y=\sum_{i=1}^{N}g_{i}(x)\cdot f_{i}(x),其中g_{i}(x)=\frac{\exp(w_{i}^{T}x)}{\sum_{j=1}^{N}\exp(w_{j}^{T}x)}$$

Feature Interactionas shown in Equation 11:

(11) $$x_{enhanced}=\alpha \cdot x+\beta \cdot MLP([img\_feat;tab\_feat])$$

The overall architecture of the mixture-of-experts classifier is as follows.

**Figure 6:**
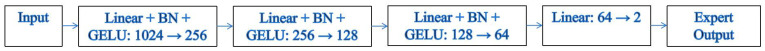
Single expert structure.

**Figure 7:**
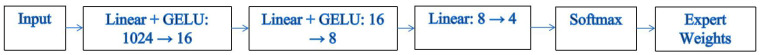
Gating network.

**Figure 8:**
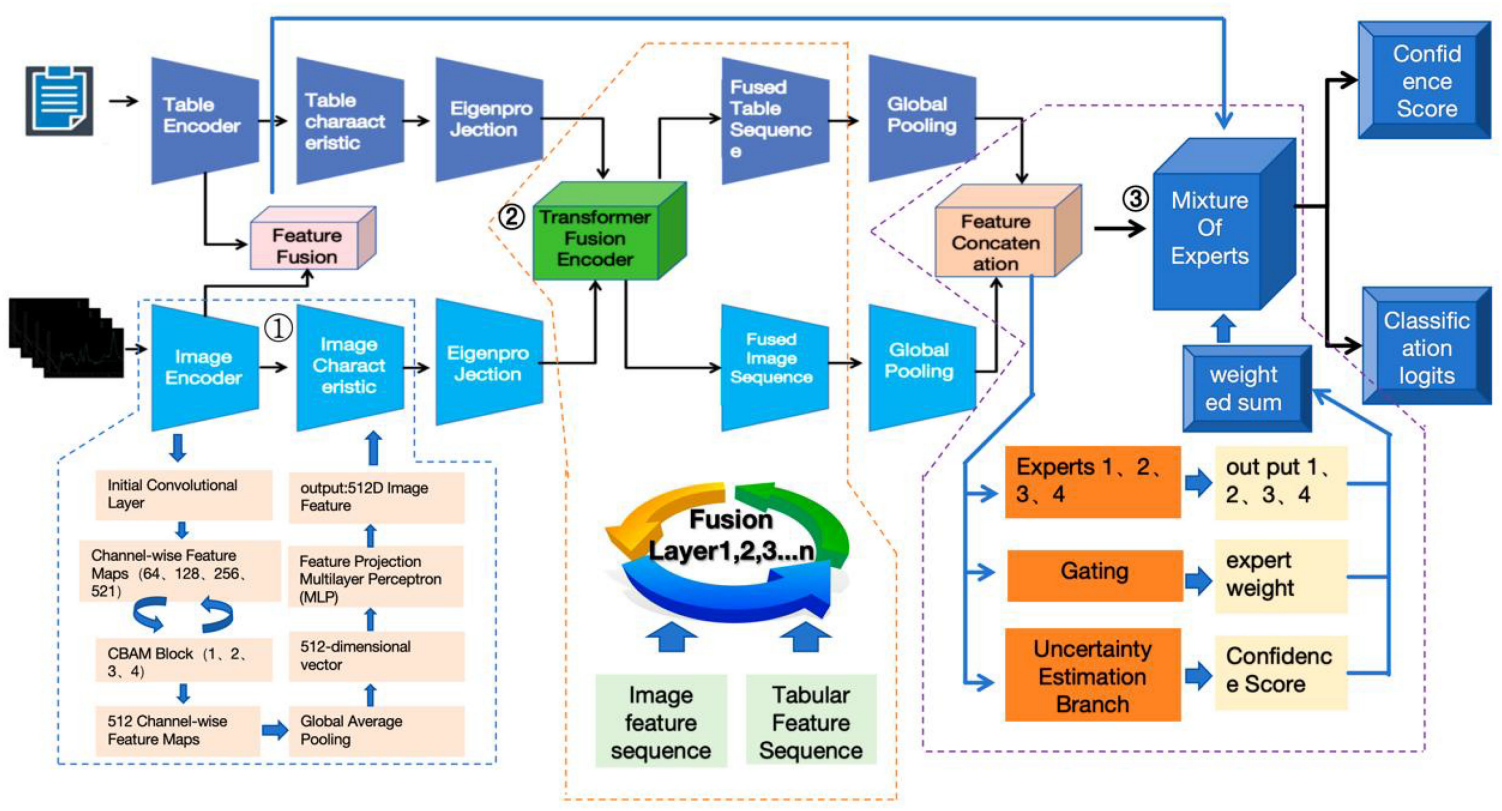
The architecture diagram of the innovative multimodal Transformer model.

Among them, the expert mechanism (**Figure 6**) and the Structure of the Gating Network (**Figure 7**) is:

To clarify the overall modeling pipeline intuitively, we present the following general flowchart (**Figure 8**):

① Image Encoder:Corresponding to **Figure 1** above, the architecture of the image encoder.
② Transformer Fusion Layer:Fusion Processing Flow of the Fusion Layer (Corresponding to **Figures 3**, **4** above).
③ Mixture Of Experts:Working Flow of Mixture-of-Experts Classifier (Corresponding to **Figures 5**–**7** above).

## 3 Results

Since the cross-validation setting can comprehensively and objectively reflect the rationality of model design and the supporting characteristics of the dataset, the above numerical trend analysis mainly focuses on the performance of the training set and validation set. In contrast, the test set contains only 7 samples. Such an extremely small sample size lacks sufficient statistical persuasiveness and cannot objectively and stably reflect the actual generalization ability of the model. Therefore, the test set is not adopted as the core basis for analysis.

### 3.1 Overall performance

After five-fold cross-validation, the model achieved a maximum accuracy of 71.43% on the fixed test set, corresponding to the model from the second fold. The precision, recall, and F1 score of this best-performing model were all 0.75, indicating balanced and stable classification performance across both positive and negative samples (**Table 1**).

**Table 1:** Model performance evaluation.

Evaluation dimensions	Evaluation metrics	Score
Classification Performance Metrics	Accuracy	71.43%
	Precision	0.75
	Recall	0.75
	F1-Score	0.75

### 3.2 Analysis of the training process

The model training curves indicate that the training loss for all folds decreases markedly with successive training rounds, and the training accuracy eventually approaches or reaches 100%, demonstrating the model’s strong learning capacity.

**Figure 9:**
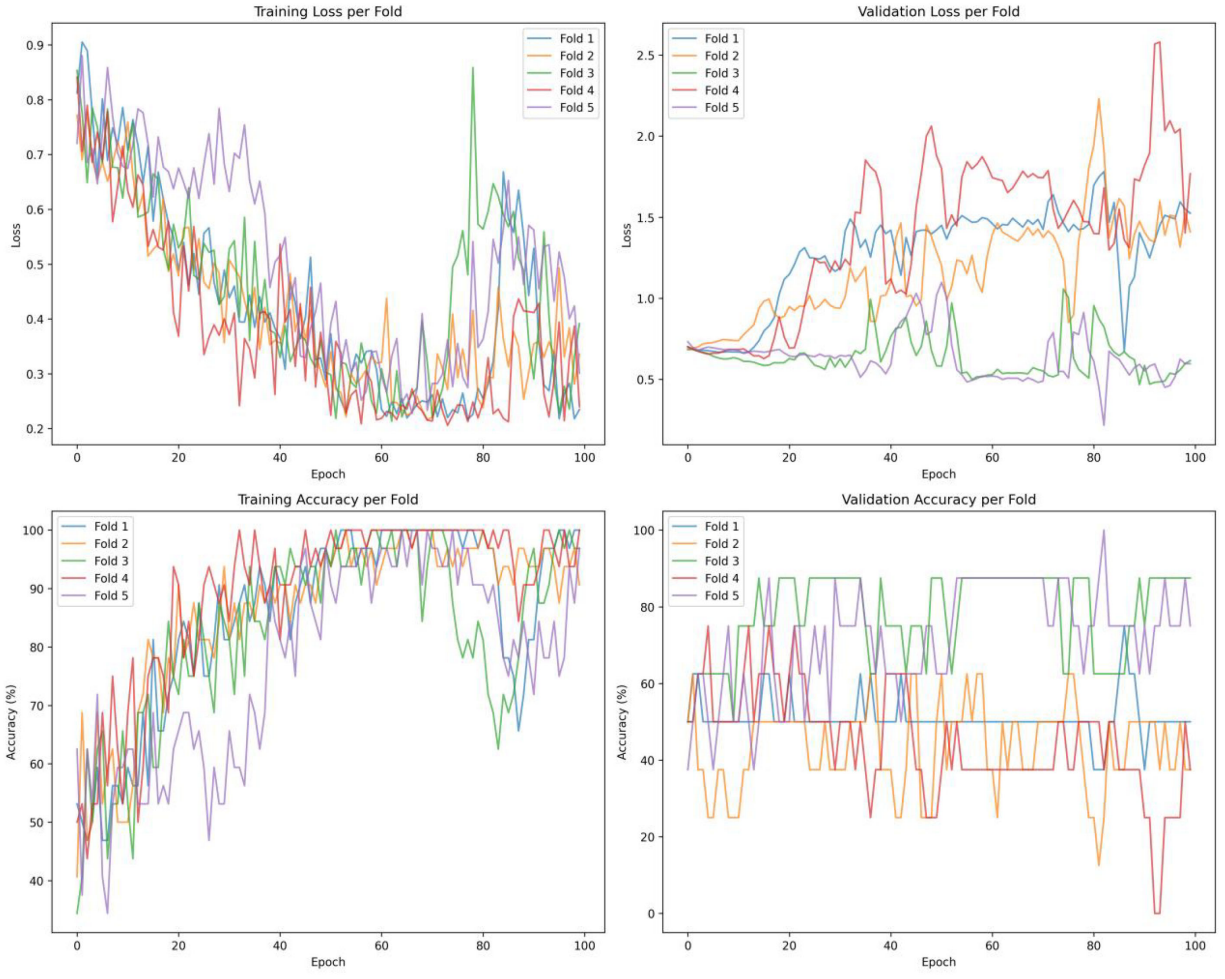
Model training curves.

However, in most folds, the validation loss either rebounds or plateaus after reaching a minimum, while the validation accuracy begins to fluctuate or decline after attaining its peak. This divergence from the continuous decrease in training loss is a typical sign of overfitting, suggesting that the model has memorized noise from the limited dataset (**Figure 9**).

### 3.3 Model stability analysis

**Figure 10:**
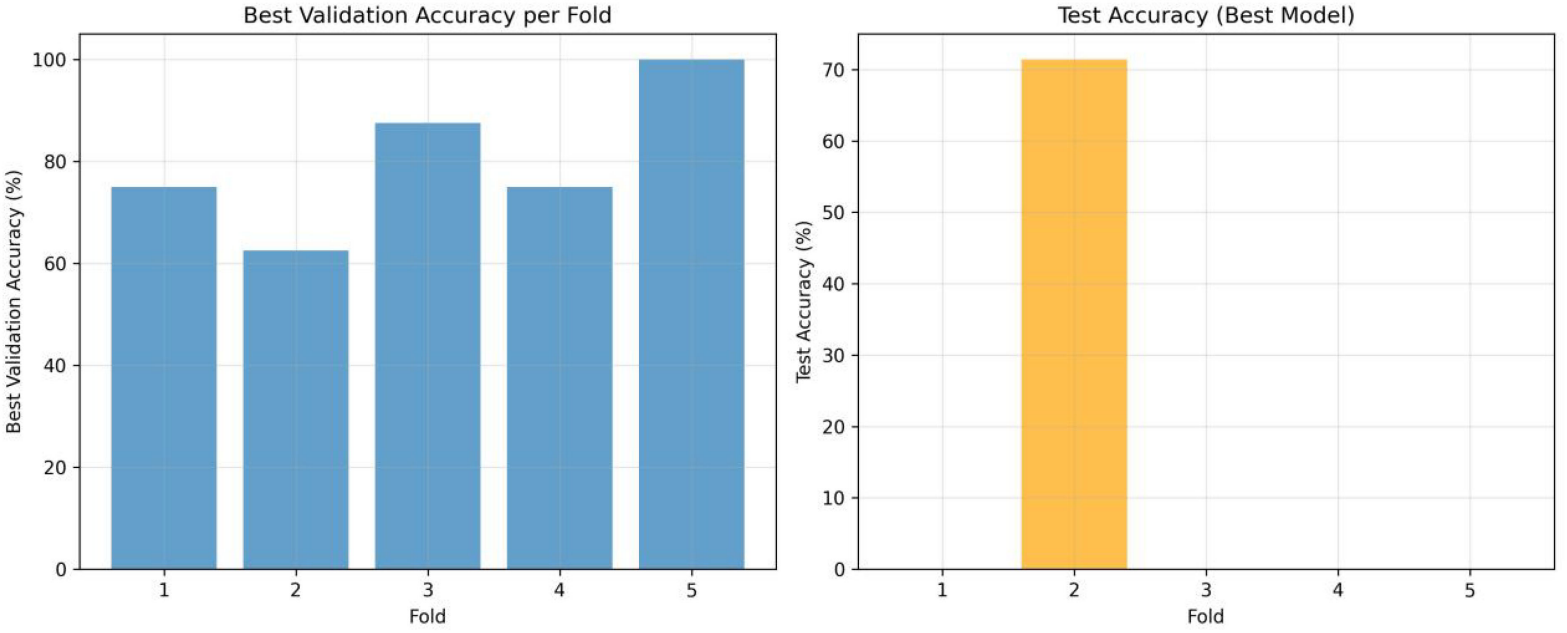
Comprehensive training curves of the model.

Performance analysis across different folds revealed notable variations in optimal validation accuracy, with a mean of 80.00% and a standard deviation of 12.75%, indicating substantial fluctuation (**Table 2**). These results highlight the model’s sensitivity to the specific partitioning of training data and underscore the challenges of maintaining stability when working with limited datasets (**Figure 10**).

**Table 2:** Model stability analysis.

Training stability metrics	
Mean fold validation accuracy	80.00%
Standard deviation of fold validation accuracy	12.75%

**Figure 11:**
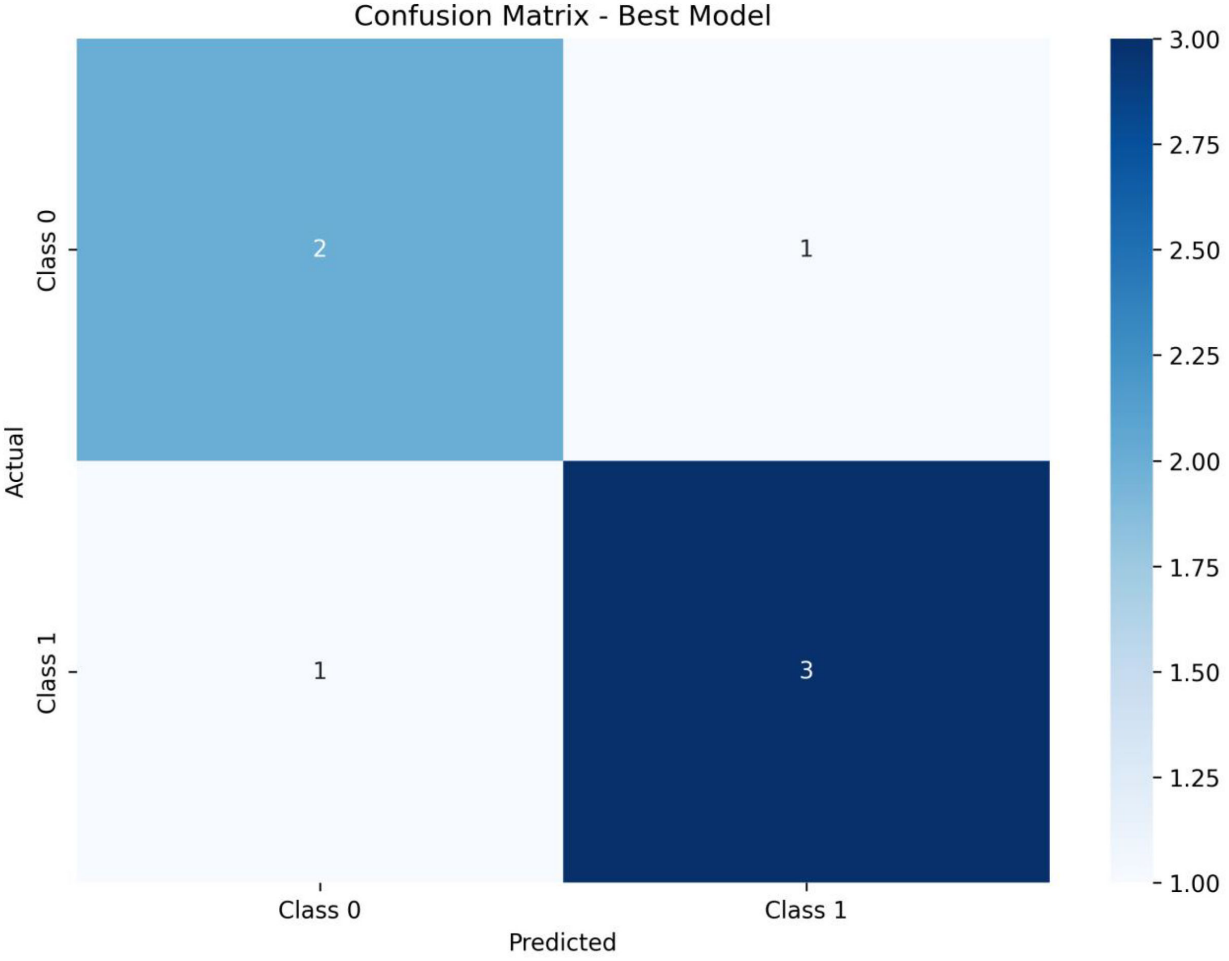
Confusion Matrix of the Optimal Model. Class 0 represents the group without auditory hallucinations; Class 1 denotes the group with verbal auditory hallucinations.

The confusion matrix of the optimal model shows that, among the seven test samples, five were correctly classified, while two were misclassified. The misclassified samples were evenly distributed across the classes (**Table 3**; **Figure 11**).

**Table 3:** Confusion matrix results.

Actual / Predicted Class	Predicted positive	Predicted negative
Actual Positive	TP=2	FN=1
Actual Negative	FP=1	TN=3

## 4 Discussion

Traditional psychiatric diagnostics primarily rely on symptomatology, with mainstream diagnostic systems emphasizing symptom persistence and functional impairment. However, the diagnostic process is often influenced by multiple factors (15, 16), and the complex variability of BD symptoms presents significant challenges to conventional diagnostic approaches. In recent years, the concept of multidimensional assessment has emerged, incorporating clinical symptoms, biomarkers, social functioning, and psychological evaluation outcomes. Machine learning, particularly deep learning models with robust autonomous neural network capabilities, has shown potential for integrating multidimensional information (17). This study proposes an innovative multimodal Transformer model and evaluates its performance in classifying ABD patients with verbal auditory hallucinations. The multimodal Transformer enables deep feature interaction and fusion across different modalities (e.g., images, text, audio) through self-attention and cross-attention mechanisms, with applications in medical image analysis (18–20), tumor classification and diagnosis (21), and psychiatric disease diagnosis, such as identifying psychiatric patients from video and audio recordings (21). For example, Guoxin Wang et al. introduced CS2former, a novel approach employing a dual-channel spatial feature extraction module within a Transformer framework, using resting-state functional magnetic resonance imaging (rs-fMRI) and T1-weighted MRI (T1w-MRI) data for BD diagnosis (18).

The experimental results of this study demonstrate that the proposed model architecture is advanced, effectively integrating heterogeneous data through mechanisms such as bidirectional cross-attention and the Ensemble of Experts classifier. The model achieved a test accuracy of 71.43% on a small dataset, demonstrating its preliminary technical feasibility. Its core biological significance lies in exploring complex, nonlinear relationships between microscopic metabolite changes and macroscopic waveform patterns, potentially reflecting overall brain energy metabolism or specific neural circuit activity states, through multimodal fusion. The model’s initial success suggests that AVHs in ABD patients may be underpinned by synergistic patterns of multiple biomarkers that can be algorithmically captured. Through multi-level, multidimensional feature interactions and attention mechanisms, this architecture effectively extracts complementary information from multimodal data, providing robust modeling capabilities for MRS data analysis.

Experimental results show that the proposed new model outperforms traditional support vector machine and random forest models in five key indicators: test accuracy, precision, recall, F1-score and best validation accuracy. It also achieves a lower validation standard deviation, delivering better generalization ability and model stability. Therefore, the new model possesses obvious effectiveness and superiority compared with mainstream benchmark models (**Table 4**).

**Table 4:** Comparative experimental results of mainstream benchmark models.

Model	Accuracy	Precision	Recall	F1	Best Val Ac	Std of best val acc
New Model	71.43%	0.75	0.75	0.75	80.00%	12.75%
SVM	68.57%	0.71	0.71	0.71	75.00%	14.28%
Random Forest	69.29%	0.72	0.72	0.72	76.67%	13.56%

According to the above results, robustness analysis suggests that the proposed model yields relatively minor result fluctuations. It tends to deliver more stable performance against dataset splitting perturbations, showing competitive classification robustness compared with the two conventional machine learning benchmark models.

Statistical difference analysis reveals a mean accuracy difference of 5.00% between the proposed model and SVM, indicating a considerable performance gap that may imply statistical significance. Similarly, a mean difference of 3.33% is observed between the proposed model and random forest, reflecting a noticeable performance advantage of the proposed method.

Statistical tests were further performed to compare the optimal validation accuracy across different models. The results preliminarily suggest that the proposed model achieves superior average performance relative to support vector machine and random forest. The observed performance improvements exhibit certain statistical implications, preliminarily supporting the rationality of the model optimization design.

Combining the standard deviation-based stability evaluation and statistical difference analysis, the proposed model presents preliminary superiority in comprehensive classification metrics, along with milder result fluctuations under data perturbation conditions and favorable robustness. Statistical verification further indicates distinguishable performance differences between the proposed model and traditional benchmark algorithms, preliminarily validating the feasibility and stable potential of the designed multimodal framework.

Several targeted optimization strategies can be adopted to mitigate validation loss fluctuations and further enhance the training stability of the proposed multimodal Transformer model. From the data dimension, expanding the scale of high-quality multimodal samples and implementing reasonable data augmentation on MRS spectral features and clinical phenotypic data may effectively enrich feature diversity and alleviate distribution bias induced by limited sample size. Meanwhile, stratified cross-validation based on clinical covariates is expected to reduce sample heterogeneity across different folds and stabilize the overall model evaluation performance. In addition, removing low-quality samples and outliers with abnormal MRS signals can help reduce noise interference during model training.

In terms of model optimization, regularization design and improved training strategies are conducive to relieving training oscillation and mitigating overfitting risks. Appropriate dropout mechanisms and weight decay settings can constrain the parameter space of the model, reducing excessive fitting to irrelevant noise and individual sample heterogeneity. The early stopping strategy based on validation loss monitoring can terminate redundant training iterations timely to avoid performance degradation caused by overfitting. Moreover, replacing conventional optimizers with the AdamW optimizer combined with cosine annealing learning rate scheduling enables decoupled weight decay and smoother gradient updates, which could effectively alleviate severe loss fluctuation in the late training stage.

Furthermore, targeted optimization of multimodal fusion modules helps narrow the model generalization gap. The introduction of adaptive modal weighting and cross-modal consistency constraints can improve the alignment between MRS metabolic features and clinical phenotypic features, suppress feature drift during model training, and facilitate the model to capture core disease-related biomarker patterns rather than superficial data correlations. Future studies will incorporate interpretable deep learning techniques to screen key discriminative features, which may further improve the training stability, cross-fold consistency, and clinical generalization performance of the multimodal model.

## 5 Limitations

In summary, the model training curves show pronounced fluctuations. The training loss drops rapidly in the early stage but then undergoes substantial ups and downs without stable convergence. The validation loss fluctuates more drastically with repeated rebounds, and the gap between training loss and validation loss is difficult to narrow. Although the training accuracy gradually approaches a relatively high level in the later stage, it remains unstable. The validation accuracy exhibits extreme fluctuations, the generalization gap mostly exceeds the reasonable threshold, and the combined score varies greatly across folds, indicating poor training stability and generalization capability.

From a data perspective, the fundamental causes of fluctuations and imbalance during training are the limited sample size and the information constraints of single-modal data. Because participants were recruited exclusively from a single tertiary-care center, selection bias may be present. A total of only 47 samples are insufficient to support the bimodal model in fully learning universally applicable discriminative patterns. Meanwhile, bimodal data combining clinical phenotypes and MRS imaging can only characterize superficial disease features and lack sufficient information dimensions to counteract interference from individual sample heterogeneity. Consequently, the model performs inconsistently across different data subsets, the training curves fail to converge steadily, and the generalization gap remains persistently large.

From a methodological perspective, this phenomenon reflects the inherent limitations of the model architecture. Although the dual-stream encoder and Transformer bidirectional cross-attention mechanism enable deep interaction of bimodal data, the absence of additional feature constraint and redundant information filtering modules makes it difficult to effectively alleviate overfitting under small-sample conditions. The model tends to capture individual noise and data distribution bias in the training set rather than the core discriminative patterns of diseases. Furthermore, without staged training and enhanced regularization strategies, the training curves present obvious fluctuations and inconsistent overall performance, which also provides a methodological direction for future model optimization.

From a biological perspective, such performance fluctuations and classification biases possess inherent rationality. The model can only capture superficial correlations between clinical phenotypes and cerebral neural metabolism. For instance, it evaluates neuronal integrity and glial activity through metabolic ratios such as NAA/Cr and Cho/Cr, yet it cannot reveal the upstream molecular regulatory mechanisms underlying hallucination symptoms.

## 6 Conclusions

This study proposes a novel multimodal Transformer model and investigates its performance in the classification task of hallucination symptoms based on MRS data. Experimental results demonstrate that the proposed model adopts an advanced architecture. With mechanisms such as bidirectional cross-attention and mixture-of-experts classifier, it can effectively fuse heterogeneous data and achieves a test accuracy of 71.43% on the small-scale dataset, which verifies its preliminary technical feasibility. In terms of application value in clinical settings, the proposed model holds promise for auxiliary differential diagnosis. It is applicable to distinguishing schizophrenia from bipolar disorder with psychotic symptoms, achieving a diagnostic accuracy of 71.43% using only MRS images and routine clinical data. With an expanded sample size in future studies, the diagnostic accuracy is expected to be further improved. This model has the potential to rapidly identify adolescent patients with bipolar depression accompanied by verbal auditory hallucinations and reduce the risk of misdiagnosis.

The core biological significance of this model lies in exploring the complex and nonlinear associations between microscopic metabolite alterations and macroscopic waveform patterns (which may reflect global cerebral energy metabolism or the activity state of specific neural circuits) through multimodal fusion. The favorable performance of the model preliminarily indicates that there may exist a synergistic variation pattern involving multiple biomarkers underlying hallucination symptoms, which can be captured by computational algorithms.

Limited by the dataset size, the current model suffers from overfitting and performance fluctuations. Nevertheless, the features adopted by the model are firmly grounded in neuroscience from a biological perspective. Its multimodal analytical strategy provides a novel data-driven perspective for understanding the complex biological mechanisms of hallucinations.

In conclusion, this study offers a promising direction for leveraging deep learning to assist biomarker discovery and symptom recognition in psychiatric disorders. Future work will focus on expanding the high-quality dataset, introducing more rigorous regularization strategies, and incorporating interpretable deep learning techniques to clarify the contribution of key features, so as to further improve the generalization ability and clinical translational value of the model.

## Data Availability

The original contributions presented in the study are included in the article/supplementary material, further inquiries can be directed to the corresponding author/s.
